# Surufatinib plus tislelizumab as later-line therapy for metastatic colorectal cancer: a single-arm, phase II trial

**DOI:** 10.1186/s12885-026-15705-z

**Published:** 2026-02-11

**Authors:** Huijun Xu, Ying Yan, JiaYu Niu, Lulu Cao, Wenju Chen, Mengge Li, Huiqin Luo, Lihong Ke, Shusheng Wu, Gang Wang, Yifu He

**Affiliations:** 1 Department of Oncology, The First Affiliated Hospital of USTC, Division of Life Sciences and Medicine, University of Science and Technology of China, Hefei, Anhui China; 2 Department of Oncology, Anhui Provincial Cancer Hospital, Hefei, Anhui China

**Keywords:** Metastatic colorectal cancer, Immunotherapy, Anti-angiogenic therapy, Tislelizumab, Surufatinib, Phase II trial

## Abstract

**Background:**

Metastatic colorectal cancer (mCRC) patients who progress following the standard chemotherapy have maintained a favourable performance status. Nevertheless, there are limited effective treatment options, particularly for a considerable proportion of tumours with a microsatellite-stable (MSS) phenotype. Based on strong preclinical evidence demonstrating the synergistic effect between an anti-PD-1 antibody, tislelizumab, and an anti-VEGFR1-3 antibody, surufatinib, we evaluated their efficacy and safety in this population of patients with treatment-refractory disease.

**Methods:**

This single-arm phase II study that recruited patients with histologically confirmed mCRC who had received ≥ 3 previous lines of systemic therapy. Critical eligibility criteria included an Eastern Cooperative Oncology Group performance status of 0 to 1 and measurable disease according to RECIST version 1.1. Patients were administered 200 mg tislelizumab intravenously every 3 weeks combined with surufatinib 250 mg orally once daily until progressive disease, intolerable toxicity, or withdrawal of consent. The primary endpoint was progression-free survival (PFS). The secondary endpoints were the disease control rate (DCR), overall survival (OS), objective response rate (ORR), and safety.

**Results:**

Sixteen patients (median age, 58 years) were enrolled between March 2022 and April 2025. The study was prematurely terminated for futility at a pre-planned ad hoc analysis. Only one patient achieved stable disease with a DCR of 6.3%, which did not meet the pre-defined threshold for continuation. The median PFS and OS were 52 days (95% confidence interval (CI): 46–73) and 157 days (95% CI: 91–not reached), respectively. The treatment-related adverse events (TRAEs) were controllable. Grade ≥ 3 TRAEs were encountered in 31.3% of patients. Hypokalaemia (31.3%), hypoalbuminaemia (50.0%), and anaemia (31.3%) were most prevalent. No treatment-related death occurred.

**Conclusions:**

Although there was a sound preclinical rationale and an urgent clinical need for effective later-line therapies of mCRC, tislelizumab combined with surufatinib demonstrated limited antitumour activity and failed to achieve the primary endpoint. This negative trial underscores the treatment difficulties associated with MSS mCRC and emphasises that immunotherapy and anti-angiogenic therapy are insufficient as monotherapies. Future studies should focus on biomarker-informed patient selection and rationally designed combination approaches to overcome resistance to immunotherapy in MSS mCRC.

**Trial registration:**

ChiCTR2200059848. Registration date: 2022/05/12. (Retrospectively registered).

**Supplementary Information:**

The online version contains supplementary material available at 10.1186/s12885-026-15705-z.

## Background

 Colorectal cancer (CRC) remains the third most common malignancy worldwide, with annual incidence rates approaching 1.9 million cases and approximately 904,000 cancer-related deaths per year [1]. Nearly 50% of patients ultimately develop metastatic CRC (mCRC), for which systemic chemotherapy—usually fluorouracil, leucovorin, and oxaliplatin (FOLFOX) or fluorouracil, leucovorin, and irinotecan (FOLFIRI)—combined with targeted therapy using anti-epidermal growth factor receptor (EGFR) or anti-vascular endothelial growth factor (VEGF) antibodies constitutes the standard first- and second-line treatment [2]. However, therapeutic options become limited following progression on these regimens. The predominant subtype of mCRC is the microsatellite-stable (MSS) disease [3], which is inherently resistant to immune checkpoint inhibitors (ICIs) owing to its low tumour mutation burden and immunosuppressive tumor microenvironment (TME) [4–6].

The combination of anti-angiogenic agents with ICIs is supported by a strong biological rationale based on synergistic mechanisms [4, 7, 8]. Angiogenesis inhibitors can normalise tumour vasculature, enhance T-cell infiltration, and reorganise the immunosuppressive TME by reducing regulatory T cells (Tregs) and myeloid-derived suppressor cells (MDSCs) [9–11]. The highly efficacious small-molecule VEGFR1-3, FGFR1, and CSF-1R-blocker surufatinib is a dual-acting agent that inhibits angiogenesis along with macrophage activity [12–14].

Concurrently, tislelizumab is a humanised anti-PD-1 monoclonal antibody designed to minimise Fc attachment and stimulate antitumour immune responses [15, 16]. Preclinical MSS CRC models using this combination have demonstrated significant tumour regression, prompting clinical trials.

The initial enthusiasm for combining immunotherapy with anti-angiogenic agents, as observed in the REGONIVO study, was attenuated by inconsistent results in subsequent confirmatory studies [17]. In North American populations, the REGONIVO approach did not replicate earlier efficacy, demonstrating an objective response rate (ORR) of 7% [18], whereas the REGOMUNE trial reported an ORR of 0% [19]. Therefore, the present phase II trial was designed to evaluate the efficacy and safety profile of this novel combination of tislelizumab and surufatinib in heavily pretreated patients with MSS mCRC to address a critical unmet clinical need.

## Methods

### Study design and participants

This prospective single-arm phase II trial (ChiCTR2200059848) was conducted at the Anhui Provincial Cancer Hospital in China between March 2022 and April 2025. The principal eligibility criteria were histologically detected mCRC; an Eastern Cooperative Oncology Group (ECOG) performance status (PS) of 0 to 1; disease progression following ≥ 3 prior lines of standard therapy (including fluoropyrimidine, oxaliplatin, irinotecan, anti-VEGF, or anti-EGFR agents); the presence of at least one measurable lesion according to RECIST v1.1; and adequate organ function.

In this study, patients with mCRC were enrolled, more than 95% of whom were presumed to have MSS or pMMR disease. Formal microsatellite instability (MSI)/MMR testing was not mandated as an inclusion criterion due to limitations in routine clinical practice at the study site; therefore, all enrolled patients were considered to have MSS disease, unless otherwise stated. Key exclusion criteria included autoimmune diseases, active brain metastases, or prior treatment with PD-1/PD-L1 blockers.

### Treatment protocol

Patients received tislelizumab 200 mg intravenously every 3 weeks and 250 mg surufatinib orally once daily. Treatment was continued until radiographic development of disease (as defined by RECIST v1.1), intolerable toxicity, withdrawal of consent, or death. Surufatinib dose could be reduced to 200 mg or 150 mg were permitted in the event of grade ≥ 3 toxicity.

### Assessments and endpoints

The main endpoint was the progression-free survival (PFS). The secondary endpoints were overall survival (OS), ORR, disease control rate (DCR), and safety, as assessed using CTCAE v5.0. All subgroup analyses (RAS/BRAF mutational status, primary tumour laterality, and presence of liver metastases) were exploratory and conducted post hoc. The study protocol did not pre-specify these analyses, and they were not registered at ChiCTR (ChiCTR2200059848); therefore, results are presented for hypothesis generation only without confirmatory inference. Tumour assessment was conducted every 6 weeks (± 7 days) using CT or MRI.

The trial was prematurely halted following 16 patients; without a pre-specified interim analysis plan. All biomarker subgroup analyses were post hoc and exploratory.

### Statistical analysis

The planned sample size was calculated to identify a hazard ratio (HR) of 0.5, corresponding to an improvement in median PFS from 3.0 months (based on historical controls in refractory MSS mCRC) to 6.0 months with 90% power at a two-sided alpha level of 0.05. Thirty-two patients were required, corresponding to a dropout rate of 10%. Although the protocol allowed for early termination based on futility, no formal statistical stopping boundary was pre-specified. The investigators conducted as ad hoc review of the observed PFS data, which ultimately led to the decision to discontinue the trial prematurely. Statistical analyses were performed using SPSS version 26.0 and R version 4.1.0. Survival distributions were estimated the Kaplan–Meier method.

## Results

### Patient characteristics

Between March 2022 and April 2025, 16 patients were enrolled and included in the efficacy and safety analyses. Table 1 summarises the baseline demographic and clinical characteristics of the patients. The median age was 58 years (range, 39–67 years), and 43.8% (7/16) of the patients were males. Most patients (87.5%, 14/16) had an ECOG PS of 1. Regarding the primary tumour site, left-sided CRC (including rectal primary tumours) predominated, accounting for 81.3% (13/16) of cases. RAS mutations were present in 50.0% (8/16) of the patients, and liver metastases in 81.3% (13/16). Thirteen patients had MSS diseases, and the MSI status was unavailable for the remaining three patients.

**Table 1:** Baseline demographics and disease characteristics

	Patient Demographics, *N* = 16
Variable	NO. of Patients (%)
Age: median [range], y	58 [39–67]
Sex	
Female	9
Male	7
ECOG PS	
0	2
1	14
Location of primary tumor site	
Left colon (includes rectum)	13
Right colon	3
*RAS* mutation status	
RAS mutated	8
RAS wild-type	8
Liver metastasis	
Yes	13
No	3
MSI status	
^a^MSS	13

Abbreviations: *ECOG* Eastern Cooperative Oncology Group, *MSI* microsatellite instability, *MSS* microsatellite stable, *RAS* rat sarcoma viral oncogene homolog

^a^ MSI status of 3 patients was not detected

### Efficacy outcomes

**Fig. 1:**
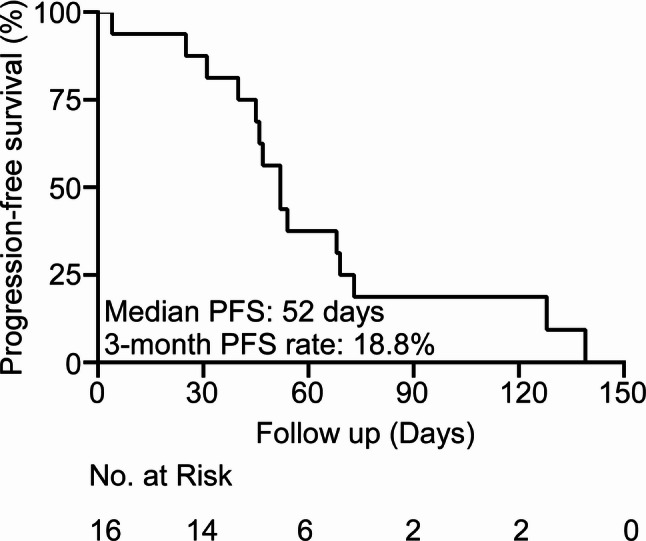
Progression-free survival of the study population

**Fig. 2:**
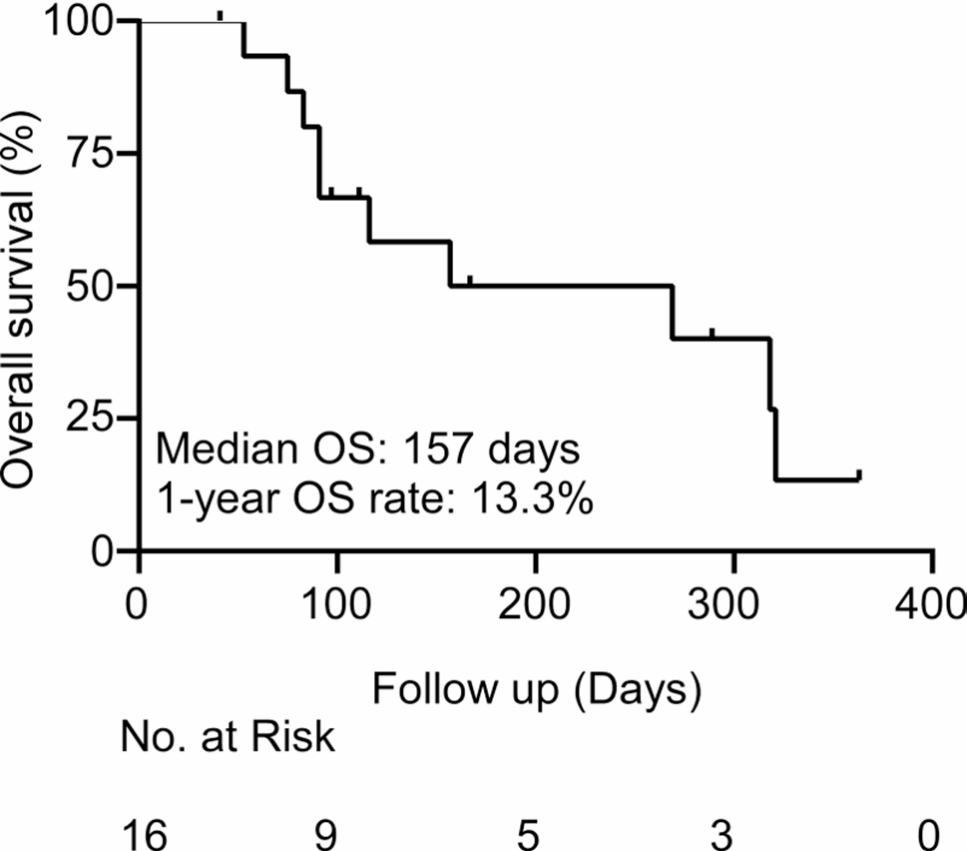
Overall survival of the study population

The trial was discontinued prematurely after the enrollment of 16 patients owing to insufficient efficacy. The median PFS was 52 days (95% confidence interval (CI): 46–73), which did not meet the pre-specified threshold of 3 months (based on historical controls of MSS CRC with median PFS 3.0 months). This threshold was derived from the target of primary endpoint (HR = 0.5; PFS from 3.0 to 6.0 months) and was not predetermined in the protocol. The analysis was performed as an ad hoc assessment (not an interim analysis). Only one patient achieved SD, corresponding to a DCR of 6.3% (1/16) and an ORR of 0%. No PR or CR were observed. The median PFS was 52 days (95% CI: 46–73 days) and its 3-month PFS rate was 18.8% (Fig. 1). The median OS was 157 days (95% CI: 91-not reached), and 1-year OS rate was 13.3% (Fig. 2).

### Subgroup analyses

**Fig. 3:**
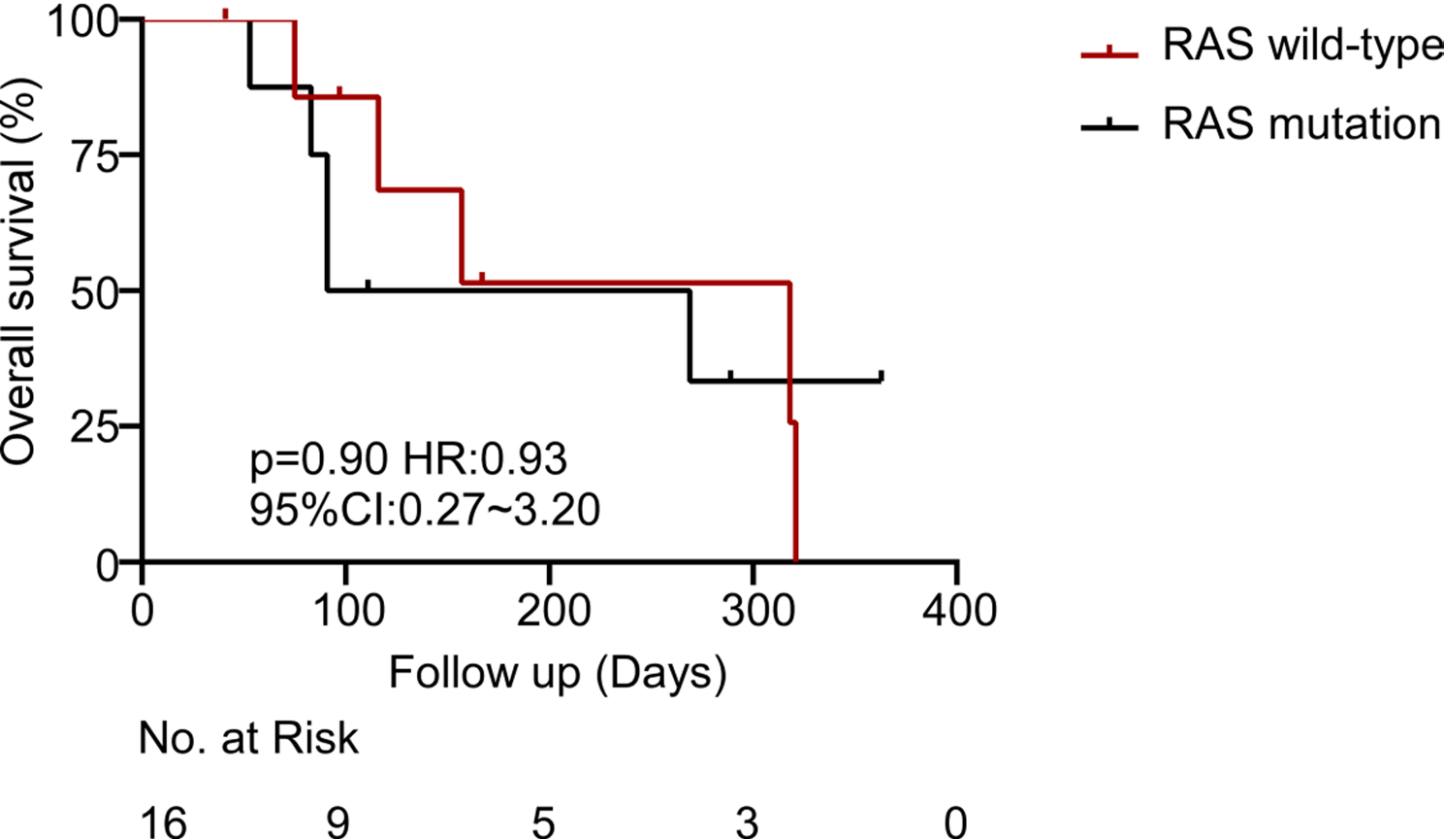
Overall survival from the date of enrollment in patients with RAS wild-type and RAS-mutated colorectal cancer

**Fig. 4:**
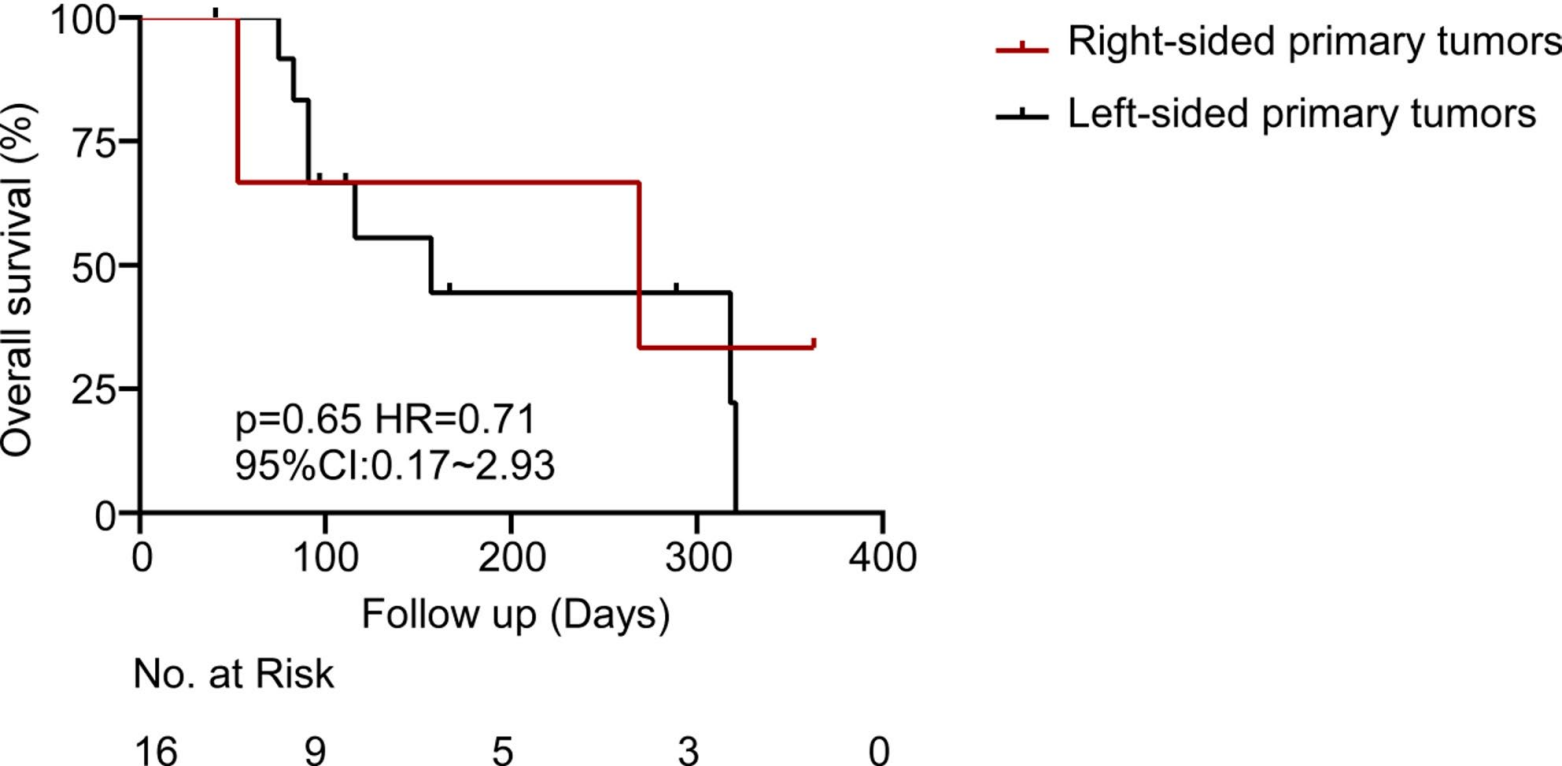
Overall survival from the date of enrollment in patients with right-sided versus left-sided primary tumors

**Fig. 5:**
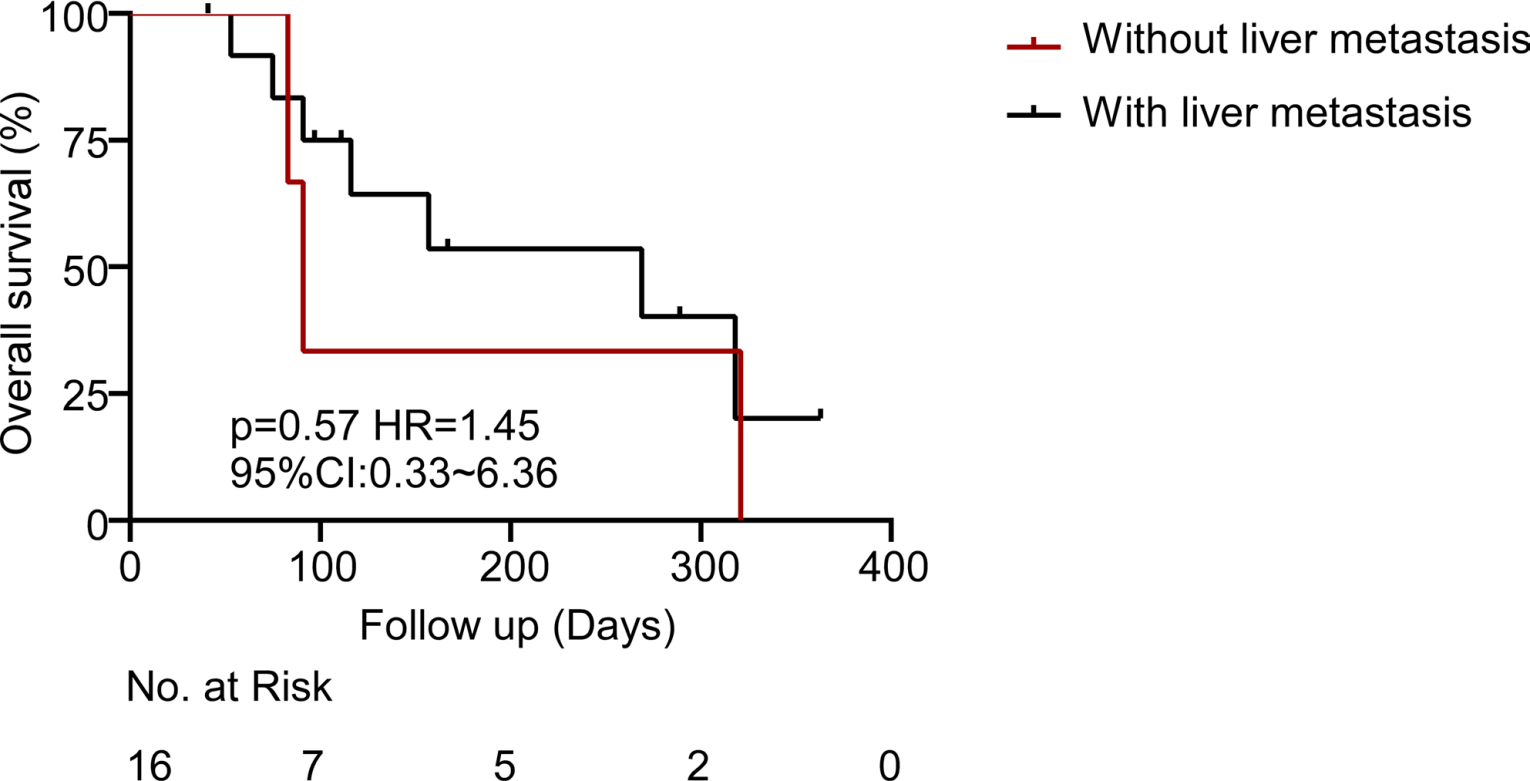
Overall survival from the date of enrollment in patients with and without liver metastasis

Exploratory subgroup analyses evaluated the OS trends according to key clinical and molecular features. Although the statistical significance was not achieved due to the small sample number, clinically notable trends were observed. RAS mutant patients and right-sided primary tumors demonstrated shorter survival as compared to left-sided or RAS wild-type tumors (Figs. 3 and 4). The most significant variation was in terms of RAS mutation status. Patients with RAS wild-type tumors exhibited a median OS that was approximately twofold in comparison with patients with RAS-mutant tumors (318 days versus 180 days; HR = 0.93, *p* = 0.90). This observation is in line with other literature demonstrating that RAS mutations have a deleterious prognostic effect on CRC [20, 21]. Interestingly, the presence of liver metastasis did not significantly impact the survival outcomes in our cohort. Moreover, the median OS of patients without liver metastases (*n* = 3) was 91 days, and was 269 days in patients with liver metastases (*n* = 13; HR = 1.45; 95% CI: 0.33–6.36; *p* = 0.57) (Fig. 5). The absence of a statistically significant difference could be explained by the small sample size in the non-liver metastasis subgroup or by the dominant influence of other prognostic factors.

### Safety profile

Treatment was generally well-tolerated and no deaths associated with treatment occurred. However, all 16 patients (100%) experienced at least one treatment-related adverse event (TRAE). Hypokalaemia (31.3%), hypoalbuminaemia (50.0%), and anaemia (31.3%) were the most common any-grade TRAEs. Grade ≥ 3 TRAE occurred in five patients (31.3%), the most prevalent ones being reduced white blood cell count, reduced neutrophil count, reduced platelet count, fatigue and increased aspartate aminotransferase levels. No Grade 4 or 5 TRAEs were observed (Table 2). TRAEs necessitated interruption of the dose in five patients (31.3%) and a reduction in the dose of surufatinib from 250 mg to 200 mg after cycle 1 due to grade 3 fatigue in one patient (6.3%).

**Table 2:** Toxic effects graded using the common terminology criteria for adverse events version 5.0

Event	Any grade	Grade 1–2	Grade 3	Grade 4–5
Hypoalbuminaemia	8(50.0%)	8(50.0%)	0	0
Anaemia	5(31.3%)	5(31.3%)	0	0
White blood cell count decreased	2(12.5%)	1(6.3%)	1(6.3%)	0
Platelet count decreased	2(12.5%)	1(6.25%)	1(6.3%)	0
Nausea	1(6.3%)	1(6.3%)	0	0
Diarrhoea	1(6.3%)	1(6.3%)	0	0
Vomiting	1(6.3%)	1(6.3%)	0	0
Hypokalaemia	5(31.3%)	5(31.3%)	0	0
Neutrophil count decreased	2(12.5%)	1(6.3%)	1(6.3%)	0
Hypocalcemia	2(12.5%)	2(12.5%)	0	0
Hypothyroidism	1(6.3%)	1(6.3%)	0	0
Fatigue	2(12.5%)	1(6.3%)	1(6.3%)	0
Creatinine renal clearance decreased	1(6.3%)	1(6.3%)	0	0
Dermatitis acneiform	1(6.3%)	1(6.3%)	0	0
Aspartate aminotransferase concentration increased	2(12.5%)	1(6.3%)	1(6.3%)	0
Elevated Alanine Aminotransferase)	1(6.3%)	1(6.3%)	0	0
Hyperbilirubinemia	1(6.3%)	1(6.3%)	0	0

## Discussion

This phase II trial represents the initial assessment of tislelizumab together with surufatinib in patients with MSS mCRC. Although there was sound mechanistic rationale to support the use of PD-1 inhibition and anti-angiogenic therapy, the regimen demonstrated limited clinical activity, with a DCR of 6.3% and a median PFS of 52 days. As a result, the study failed to achieve its primary endpoint and was terminated early.

The lack of clinical synergy suggests three mechanistic barriers in MSS mCRC immunotherapy that remained unaddressed. First, profound refractoriness of the patient population: all patients had undergone ≥ 3 previous lines of therapy (median 4 lines, range 3–7), which is associated with irreversible T-cell exhaustion and impaired immune reconstruction [22, 23]. These patients typically harbor accumulated resistance mechanisms that may diminish the efficacy of subsequent regimens including those involving immunotherapy-based combinations.

Second, the unfavorable clinical outcome may be attributed to the immunosuppressive TME of MSS mCRC. MSS CRCs typically exhibit low rates of CD8^+^ T-cell infiltration, high Treg density and the up-regulation of other immune checkpoints such as TIM-3 and LAG-3 which may confer resistance to PD-1 block and VEGF block alone [24]. This immunologically quiescent microenvironment inherently limits the efficacy of PD-1 blockade, as demonstrated by the 0% ORR in the REGOMUNE trial [19]. Importantly, we now attribute this to immunomodulatory properties of surufatinib: daily continuous dosing suppresses NK cell activity and promotes Treg expansion [25], which directly counteracts the effect of Tislelizumab. This mechanism may explain the similarly poor ORR observed in our study and the REGOMUNE trial, in contrast to fruquintinib-based combinations, which achieved higher ORRs (20.9%; NCT03903705) and employed intermittent dosing schedules that better preserved immune function [26].

Third, dosing and scheduling of surufatinib may have compromised the results obtained. Continuous use of high-dose surufatinib (250 mg/day) may cause vascular pruning leading to impaired T-cell traffic and infiltration of tumor tissues [25]. Preclinical models have suggested that alternative dosing regimens, including intermittent dosing schedules or reduced daily doses, may better maintain vascular normalisation and immune cell recruitment to facilitate synergy with ICIs [27–29]. The failure of our regimen to implement this strategy contributed to a lack of synergy. Collectively, these findings underscores the fact that successful MSS mCRC immunotherapy requires less refractory patients, MSS tumours with modifiable immunomodulatory dosing schedules.

Although our analysis centred on clinical and genomic biomarkers (RAS/BRAF, tumour sidedness, and liver metastases), the lack of immune-related biomarkers (PD-L1, TMB, and T-cell infiltration) limited our ability to outline the mechanisms of immune resistance in MSS CRC. Further prospective studies should mandate centralised or locally validated MSI/MMR testing before enrollment. The spatial heterogeneity of T-cell infiltration has been implicated in primary resistance to PD-1 inhibitors in MSS tumours [30]. In our efficacy analysis, we identified three patients with unknown MSI/MMR statuses. Although the prevalence of MSI-H disease in unselected mCRC is low (4–5%) [3], misclassification cannot be completely excluded. Nevertheless, considering the overall clinical behaviour, pattern of treatment response, and high baseline probability of MSS disease, any such misclassification is unlikely to meaningfully affect the study’s conclusions. Advanced approaches such as multiplex immunohistochemistry and spatial transcriptomics should be incorporated into future studies to characterise the immunosuppressive TME in patients with refractory MSS CRC.

The fact that our subgroup analyses are post hoc in nature reflects the practical limitations of ultra-early-phase trials in extremely refractory populations. These analyses should be regarded as signal-generating not predictive, especially given the limited availability of tissue and statistical power. In summary, the tislelizumab and surufatinib combination possesses sound mechanistic rationale; however, its clinical efficacy in refractory MSS mCRC is limited. Future research must address patient selection via biomarkers, optimized dosing schedules, and involve other agents targeting complementary immune resistance pathways.

A numerically favorable, albeit statistically non-significant, trend was observed toward improved OS in patients with RAS wild-type tumors compared to those with RAS mutations (median OS: 318 days vs. 180 days; 95% CI: 0.27–3.20; *p* = 0.90). This observation is mechanistically consistent with the established role of oncogenic RAS signaling in the formation of immunosuppressive TME [31–34]. Preclinical and clinical studies have demonstrated that MDSCs are actively recruited in RAS-mutant cancers and represent an important population which suppresses antitumor T-cells and promotes immunotherapy resistance [35–39]. Hence, the observed survival difference, although statistically insignificant, may be attributed to the failure of patients with RAS mutations to develop an efficient antitumor immune response due to MDSC-driven immunosuppression coupled with the limited size of the current cohort.

This trial underscores the ongoing difficulty in applying immunotherapy to MSS mCRC, a tumor type that is well known to be resistant to ICIs. Current biomarker evaluations through PD-L1, tumor mutational burden, and MSI have proven inadequate for patient stratification, thereby necessitating the urgent need to explore additional predictive biomarkers. Newer candidates, like transforming growth factor-beta (TGF-β) signatures and tertiary lymphoid structures, may yield more information on the tumor immune microenvironment [40, 41]. Moreover, rational combination strategies including triple-agent regimens with ICIs, anti-angiogenic agents, and chemotherapy or TGF-β blockers show potential for overcoming both primary and adaptive resistance mechanisms. Importantly, shifting immunotherapeutic interventions to earlier lines of treatment when host immunity remains more robust and the TME less suppressive, may enhance immune priming and clinical outcomes. Future research should emphasize comprehensive characterization of the immune microenvironment to identify possible responsive subsets within this heterogeneous population.

The small sample size (*n* = 16) may account for the inability to detect a clinically meaningful signal, as the phase I trial of surufatinib/toripalimab (NCT03879057) which recruited four patients with CRC demonstrated one PR and three SDs. Multicenter collaboration in such trials should be considered to speed up the enrollment in this highly refractory population.

## Conclusion

The combination of tislelizumab and surufatinib exhibited acceptable toxicity but limited antitumor activity in heavily pretreated patients with MSS mCRC. This negative trial underscores the therapeutic resistance in this population and suggests that dual blockade of PD-1 and VEGF pathways alone proves inadequate to achieve meaningful clinical benefit. Future investigations must prioritize biomarker-guided patient selection and mechanism-based triple-combination regimens to overcome immune resistance in MSS CRC.

## Supplementary Information

Supplementary Material 1.

## Data Availability

The data from the study is available from the corresponding author on reasonable request.

## Abbreviations

CI: Confidence Interval
CRC: Colorectal Cancer
DCR: Disease Control Rate
ECOG: Eastern Cooperative Oncology Group
EGFR: Epidermal Growth Factor Receptor
FOLFIRI: Fluorouracil, leucovorin, and irinotecan
FOLFOX: Fluorouracil, leucovorin, and oxaliplatin
HR: Hazard Ratio
ICIs: Immune Checkpoint Inhibitors
mCRC: Metastatic Colorectal Cancer
MDSCs: Myeloid-derived suppressor cells
MSI: Microsatellite instability
MSS: Microsatellite-stable
ORR: Objective Response Rate
OS: Overall Survival
PFS: Progression-free survival
PS: Performance Status
TGF-*β*: Transforming growth factor-beta
TMB: Tumour mutation burden
TME: Tumour microenvironment
TRAEs: Treatment-related adverse events
Tregs: Regulatory T cells
VEGF: Vascular endothelial growth factor
