# Supplementary material for: Surufatinib plus tislelizumab as later-line therapy for metastatic colorectal cancer: a single-arm, phase II trial
Source: BMC Cancer. 2026 Feb 11;26:360. doi: 10.1186/s12885-026-15705-z (PMC12997721; doi:10.1186/s12885-026-15705-z)
Supplement: Supplementary file 1 — Supplementary Material 1. [file 12885_2026_15705_MOESM1_ESM.pdf]

editage

Editing Certificate

This document certifies that the manuscript listed below has been edited to ensure language and grammar accuracy and is error free in these aspects. The logical presentation of ideas and the structure of the paper were also checked during the editing process. The edit was performed by professional editors at Editage, a brand of Cactus Communications. The author's core research ideas were not altered in any way during the editing process. The quality of the edit has been guaranteed, with the assumption that our suggested changes have been accepted and the text has not been further altered without the knowledge of our editors.

MANUSCRIPT TITLE

...

AUTHORS

Huijun Xu

ISSUED ON

January 17, 2026

JOB CODE

XKGQU_2

THE EDITAGE Promise QUALITY GUARANTEE

Prabh Grewal
Senior Vice President - Editage

editage | helping you get published

Since 2002, Editage has helped over 430,000 authors publish around 1.2 million research papers in scholarly journals across over 1000 disciplines through editorial, translation, transcription, and publication support services. Editage is a brand of Cactus Communications (cactusglobal.com), a science communication and technology company.

CACTUS.

GLOBAL :
+1(669) 272-1214 | request@editage.com

CHINA :
400-001-8237; 021-60209400 | fabiao@editage.cn
